# Beringia and the peopling of the Western Hemisphere

**DOI:** 10.1098/rspb.2022.2246

**Published:** 2023-01-11

**Authors:** John F. Hoffecker, Scott A. Elias, G. Richard Scott, Dennis H. O'Rourke, Leslea J. Hlusko, Olga Potapova, Vladimir Pitulko, Elena Pavlova, Lauriane Bourgeon, Richard S. Vachula

**Affiliations:** ^1^ Institute of Arctic and Alpine Research, University of Colorado, Boulder, CO 80309, USA; ^2^ Department of Anthropology, University of Kansas, 622 Fraser Hall, 1415 Jayhawk Blvd, Lawrence, KS 66045, USA; ^3^ Department of Anthropology, University of Nevada-Reno, 1664 N. Virginia Street, Reno, NV 89557, USA; ^4^ Human Evolution Research Center, University of California-Berkeley, 3101 Valley Life Sciences Building, Berkeley, CA 94720-3140, USA; ^5^ Centro Nacional de Investigación sobre la Evolución Humana (CENIEH), Burgos, Spain; ^6^ Pleistocene Park Foundation, Philadelphia, PA 19006, USA; ^7^ Department of Mammoth Fauna Studies, Academy of Sciences of Sakha, Yakutsk, Russia; ^8^ The Mammoth Site of Hot Springs, Hot Springs, SD 57747, USA; ^9^ Institute of the History of Material Culture, Russian Academy of Sciences, Dvortsovaya nab., 18, 191186 St Petersburg, Russia; ^10^ Peter the Great Museum of Anthropology and Ethnography (Kunstkamera), Russian Academy of Sciences, 3, Universitetskaya nab., St Petersburg 199034, Russian Federation; ^11^ Arctic and Antarctic Research Institute, Russian Federal Service for Hydrometeorology and Environmental Monitoring, 38 Bering Street, 199397 St Petersburg, Russia; ^12^ Kansas Geological Survey, University of Kansas, 1930 Constant Ave., Lawrence, KS 66047, USA; ^13^ Department of Geosciences, Auburn University, 2050 Beard Eaves Coliseum, Auburn, AL 36849-5305, USA

**Keywords:** Beringia, Quaternary, palaeogenomics, archaeology, migration

## Abstract

Did Beringian environments represent an *ecological barrier* to humans until less than 15 000 years ago or was access to the Americas controlled by the spatial–temporal distribution of North American ice sheets? Beringian environments varied with respect to climate and biota, especially in the two major areas of exposed continental shelf. The East Siberian Arctic Shelf (‘Great Arctic Plain’ (GAP)) supported a dry steppe-tundra biome inhabited by a diverse large-mammal community, while the southern Bering-Chukchi Platform (‘Bering Land Bridge’ (BLB)) supported mesic tundra and probably a lower large-mammal biomass. A human population with west Eurasian roots occupied the GAP before the Last Glacial Maximum (LGM) and may have accessed mid-latitude North America via an interior ice-free corridor. Re-opening of the corridor less than 14 000 years ago indicates that the primary ancestors of living First Peoples, who already had spread widely in the Americas at this time, probably dispersed from the NW Pacific coast. A genetic ‘arctic signal’ in non-arctic First Peoples suggests that their parent population inhabited the GAP during the LGM, before their split from the former. We infer a shift from GAP terrestrial to a subarctic maritime economy on the southern BLB coast before dispersal in the Americas from the NW Pacific coast.

## 1. Introduction

Recent palaeogenomics research has confirmed the hypothesis, proposed more than two centuries ago, that the Indigenous peoples of the Western Hemisphere are derived from a population in Northern Asia. Palaeogenomics also confirms the thesis that the ancestral Native American population migrated from Asia to North America via the Bering Strait region, where large areas of continental shelf were exposed during periods of cold climate by lowered sea level [1,2].

**Figure 1.**
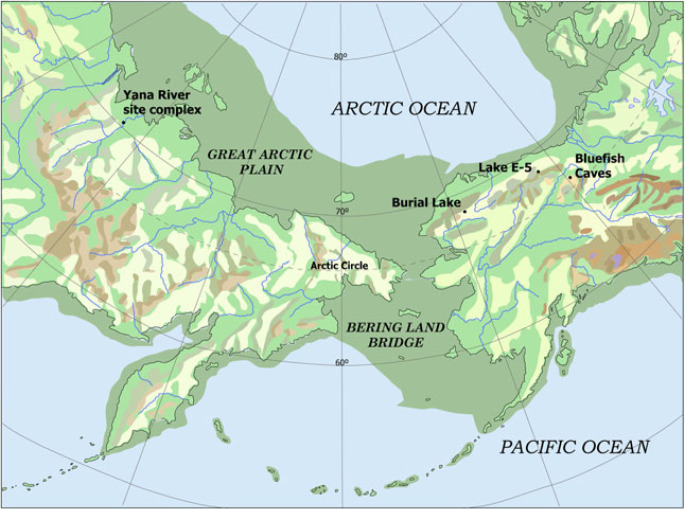
Map of Beringia, showing location of sites mentioned in the text.

In 1937, Eric Hultén proposed the palaeogeographic label *Beringia* for the exposed shelf areas in the Bering Strait region, which he hypothesized, based on modern plant distribution, had provided a refugium for arctic and subarctic plants during the cold-climate periods [3]. There are two such areas of continental shelf, both of which now are fully submerged. One is the *Bering-Chukchi Platform*, which adjoins Chukotka and western Alaska and often is equated with the ‘Bering Land Bridge' (BLB). The other is the *East Siberian Arctic Shelf*, which extends from the Chukchi Sea to the western shore of the Laptev Sea and the Taimyr Peninsula (i.e. across the central and eastern Siberian Arctic). Although all the Bering-Chukchi Platform falls within the geographical definition of Beringia, the East Siberian Arctic Shelf occupies a larger region that lies outside both the original and later, expanded definitions of Beringia [4,5] (figure 1).

In this review, we address the question of the role of Beringia in the peopling of the Western Hemisphere. The Beringian environment often has been viewed as the critical variable in the timing of migration(s) from Northern Asia to the Americas. Specifically, Beringia is widely seen as having represented an *ecological barrier* to human populations due to cold-climate effects on plant and animal productivity. According to this view, climate warming during the final millennia of the Pleistocene or after 15 000 years ago (15 ka) rendered the exposed Bering-Chukchi Platform habitable for humans, allowing movement of a North Asian population into Alaska and rapid dispersal throughout the Western Hemisphere [6,7]. An alternative view is that the spatial and temporal pattern of deglaciation after 19 ka controlled the timing and routes of human dispersal in mid-latitude North America and farther south.

The Beringian environment is linked to another major problem in the peopling of the Western Hemisphere: explaining the primary source of biological variation among living First Peoples. The principal divisions among Native Americans have been explained as a product of either (a) multiple migrations from Northern Asia to the Americas or (b) regional diversification of a single population after its arrival in the Western Hemisphere. A multiple migration thesis was proposed in 1986 by Greenberg *et al*. based on identification of three major linguistic and biological subdivisions (‘Eskimo-Aleut,’ Na-Dene and ‘Amerind’) among First Peoples [8]. Their ‘three-wave model’ was resuscitated in 2012 by Reich *et al*. based on the first whole-genome analysis of the Native American population [9].

Alternatively, Szathmary and others argued that Native American population biology was more parsimoniously explained by a single migration from Asia (before the Last Glacial Maximum (LGM), which began 26.5 ka) and subsequent diversification in the Western Hemisphere [10,11]. This view was supported by mtDNA studies of living populations during the 1990s suggesting an early divergence of ancestral First Peoples from their East Asian source population [11,12]. In 2007, Tamm *et al*. proposed, also based on mtDNA analyses, that the Native American founder population had been isolated in Beringia during the LGM (‘Beringian standstill’ model) [13]. The standstill model rested on the assumption that Beringian environments represented a potential refugium—not an ecological barrier—for ancestral First Peoples before their dispersal throughout the Western Hemisphere about 15 ka [14,15].

## 2. Climate, sea level and glaciation

### (a) Before the Last Glacial Maximum (59–26.5 ka)

A new history of global sea level recently has been proposed for the period preceding the LGM (Marine Isotope Stage 3 or MIS 3 [59–29 ka]), based on dated beach ridges on the Atlantic coast of North America and sediment cores from East Asia. Pico *et al.* postulate significantly higher sea level than previously thought for this period, peaking at 40 m below that of the present day approximately 45 ka [16]. This implies a reduced volume of continental ice, and the proposed revision of sea-level history has been applied to reconstruction of a smaller *Laurentide ice sheet* (LIS) in Canada during 45–30 ka [17,18].

Higher sea levels and a reduced LIS during the period preceding the LGM have implications for human settlement of Beringia and the Western Hemisphere. As sea level rose above −53 m, the Bering-Chukchi Platform would have been flooded, severing a land connection between Asia and North America. A smaller LIS during MIS 3 would have provided a wider *ice-free corridor* between the LIS and the *Cordilleran ice sheet* in the Canadian Rockies, enhancing interior access from Beringia to mid-latitude North America before the LGM. It also implies a slower and later closing of the corridor, as both ice sheets expanded and eventually coalesced in response to colder climates after 40 ka (i.e. providing prolonged access to mid-latitude North America).

**Figure 2.**
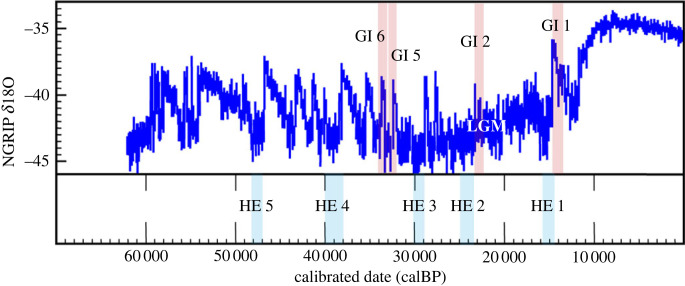
Palaeoclimate record for the past 60 000 years, based on the NGRIP oxygen-isotope core, calibrated with the IntCal20 radiocarbon calibration curve [21], showing Heinrich Events 5–1 (HE 5–HE 1) and interstadials mentioned in the text (GI 6, etc.) (adapted from OxCal v4.4.4 https://c14.arch.ox.ac.uk/oxcal/OxCal.html).

The revised sea-level history and reconstruction of the LIS have been disputed, based on both geochronology and climate-stratigraphy, and the issue currently is subject to debate among Quaternary geologists [19,20]. As a result, there is uncertainty about the presence of a land connection between Asia and North America and the spatial-temporal dimensions of the ice-free corridor before the LGM. There is agreement however between the revised and earlier proposed sea-level histories that global sea level fell rapidly after 40 ka (i.e. the beginning of cold *Heinrich Event 4* or HE 4) reaching a minimum of at least −120 m at the beginning of the LGM (approx. 26.5 ka) [16,20]. There also is consensus that re-advance of the LIS and Cordilleran ice sheet began during HE 4, eventually blocking both interior (i.e. ice-free corridor) and NW Pacific coastal access from Beringia to mid-latitude North America [17,18,20] (figure 2).

### (b) The Last Glacial Maximum (26.5–19 ka)

There currently is less uncertainty about sea-level history and glacial chronology for the LGM, although the timing of the coalescence of the LIS and Cordilleran ice sheet remains unclear. While Dalton *et al.* [17] concluded that the ice-free corridor closed during the early LGM (≥ 25 ka) bison genetics suggest that it may have remained open as late as 23 ka [22]. Also relevant to Beringia and its role in the peopling of the Western Hemisphere is the discovery that the Verkhoyansk Mountains were not glaciated during the LGM [23]. Because these mountains extend above the Arctic Circle, where they sometimes exceed 1900 m in elevation, the absence of glaciers 26.5–19 ka can be explained only by limited moisture supply, underscoring the extreme aridity of the region (East Siberian Arctic Shelf) during the LGM (discussed below).

### (c) After the Last Glacial Maximum (19–11 ka)

New dates are available for both the deglaciation of the NW Pacific coast and the reopening of the interior ice-free corridor in western Canada. Deglaciation of the NW Pacific coast began as early as 18 ka and a viable coastal route between southern Beringia and mid-latitude North America may have been available by 17 ka (although probably requiring some use of watercraft) [24]. Also pertinent to human settlement is early retreat of the ice on the southern coast of Alaska (less than 18 ka), which would have facilitated movement from the southern BLB to the NW Pacific coast before the rapid rise in sea level (less than 16 ka) [25].

The reopening of the corridor between the Cordilleran ice sheet and LISs now appears to have taken place after 14 ka. A narrow ice-free corridor apparently extended from the Yukon to southern Alberta (roughly 2000 km in length) at 13.8 ± 0.5 ka and might have allowed limited movement of people from the interior of Beringia to the Northern Plains at this time (although movement of bison through the corridor is thought to have occurred less than 13.4 ka) [22,26]. The late dating of the post-LGM ice-free corridor indicates that the rapid dispersal of First Peoples in mid-latitude North America that began before 14.5 ka (based on a group of reliably dated archaeological sites in mid-latitude North America) probably began on the NW Pacific coast, which was largely deglaciated at this time [2,24,25].

## 3. Reconstructing Beringian environments

### (a) Bering-Chukchi platform (‘Bering Land Bridge’)

Debate and controversy have attended the reconstruction of BLB environments for many decades. Hultén postulated a refugium for ‘oceanic plants’ in the southern region of Beringia based on the distribution of living taxa [3]. During the 1960s, Colinvaux analysed pollen cores from islands in the Bering Sea and localities in western Alaska, concluding that the BLB supported an ‘herbaceous tundra like that of modern Barrow’ although possibly with some ‘steppe characteristics’ [27]. Guthrie disputed this reconstruction, based on the analysis of the mammalian fauna, which suggested a northern grassland (i.e. exhibited a predominance of grazers, such as bison and horse) [28].

During the 1990s, the analysis of cores retrieved from the former surface of the land bridge indicated a mesic tundra, and Guthrie and others later proposed that the low-lying BLB supported a mesic tundra belt that acted as an ecological barrier to at least some of the steppe-adapted taxa (including invertebrates) on opposing sides of the Bering Strait [29–31]. Both the oceanic influence of the North Pacific on the southern land bridge and poor drainage conditions created by the lack of topographic relief on the BLB would have promoted a mesic tundra environment. The debate has continued, nevertheless. Several researchers postulated an LGM refugium for some arboreal taxa on the land bridge (based on pollen data) [32]. New palaeo-botanical studies (including ancient DNA data) on one of the Pribilof Islands indicated that this was unlikely, however, at least during the post-LGM period [7,33].

**Figure 3.**
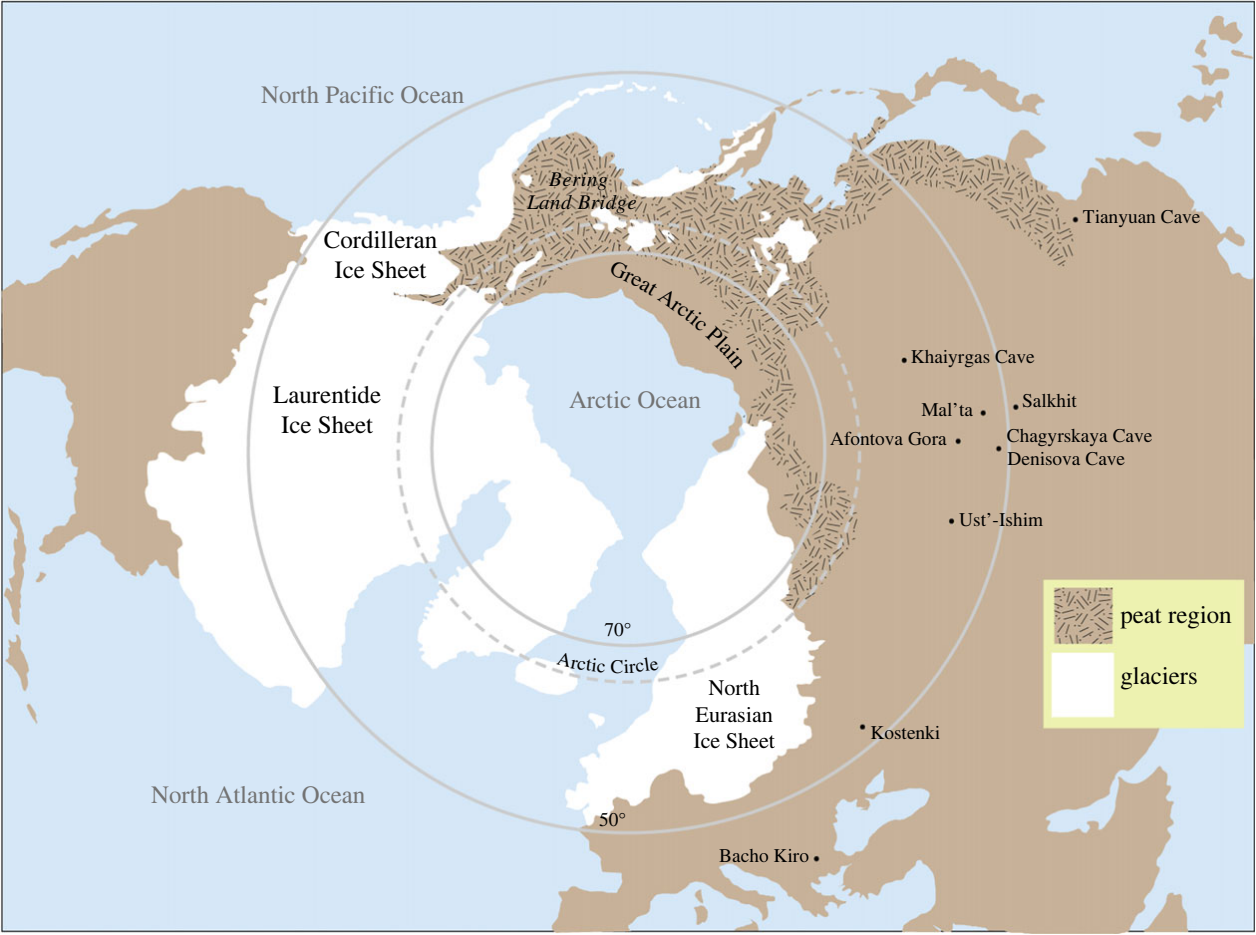
Glaciation in the Northern Hemisphere during the LGM, showing the extent of the peat region and location of sites in northern Eurasia mentioned in the text (adapted from Lindgren *et al*. fig. 1 [34]).

Regardless of the net primary productivity (NPP) levels during the LGM and later, terrestrial habitat on the land bridge probably had a low carrying capacity for humans. Even at the most southerly latitudes of the BLB, virtually none of their diet would have been derived directly from plant foods, while the terrestrial mammalian biomass for mesic tundra probably was lower than that of high-latitude steppe-tundra of the LGM and post-LGM (including arctic Beringia, as described below). Many large grazers, such as bison and horse, that inhabited arctic Beringia may have been rare or absent on the southern land bridge, where extensive peat formation took place during the LGM [34], although mammoth, which survived after its inundation on one of the Pribilofs, was present. A seasonal resource in the form of migratory waterfowl, attracted to ponds and wetlands on the poorly drained BLB, also may have existed [35] (figure 3).

### (b) East Siberian Arctic Shelf (‘Great Arctic Plain’)

By contrast, there is a consensus regarding the climate and biota of the exposed East Siberian Arctic Shelf, which supported a dry steppe-tundra biome populated by a diverse large mammalian fauna. The critical variable was the lack of moisture, intensified during intervals of extreme cold (*cryo-aridity*), as falling sea levels moved the Arctic Ocean coast hundreds of kilometres north and the north Eurasian ice sheet reduced the flow of moisture from the west [4]. As a result, conditions were too dry for formation of the wet tundra soils and peat bogs found in the Arctic today. In their place, warmer and more productive soils, similar to those found in the northern steppe zone, developed across the exposed shelf areas, as well as over the adjoining lowland basins of northeastern Siberia (e.g. Kolyma Basin) and the North Slope of Alaska. Schirrmeister *et al*. labelled this landscape the ‘Great Arctic Plain’ (GAP) [31,36,37].

Recent efforts to reconstruct the vegetation of the GAP, described as ‘steppe-tundra’ or ‘cryo-arid steppe’ without modern analogue, have drawn from a large palaeogenomic plant database extracted from sample localities across the circumpolar zone. The vegetation was dominated by forbs, graminoids and willow shrubs, reaching a peak in diversity at the beginning of the LGM (26.5 ka), followed by a marked decrease in diversity during the coldest phases of the LGM [38]. The mammalian fauna, reconstructed from hundreds of radiocarbon-dated bones recovered from localities on the northeastern Siberian lowlands, arctic Alaska/Yukon and islands in the East Siberian Sea, included typical northern steppe dwellers, such as bison, horse and woolly rhinoceros, along with tundra species, such as reindeer and musk ox. Woolly mammoth, which enjoyed a wide dietary niche, was especially common and survived on island remnants of both the GAP and southern BLB into the mid Holocene [4,31,36,39].

The large mammal populations apparently fluctuated significantly, probably in response to climate change and its effects on plant productivity. Major changes in population size are inferred from variations in numbers of radiocarbon-dated bones, as well as from genetic bottlenecks in the ancient DNA data. Mann *et al*. hypothesized that populations peaked at the beginning of interstadials when plant productivity rose in response to increased temperature and moisture, but before wet tundra soils could develop [36]. Several researchers report a major decline in the mammoth population at the end of the LGM (*ca* 20–15 ka), while bison numbers seem to decline sharply in eastern Beringia during the same period [36,40]. It currently is unclear what relationship these fluctuations may have had to any human occupants of arctic Beringia.

## 4. Human settlement of northern Eurasia

The settlement of latitudes above 45° North in Eurasia by anatomically modern humans and their predecessors provides an essential context for understanding the peopling of Beringia and the Western Hemisphere. Although Neanderthals and Denisovans adapted to some cold and relatively unproductive (i.e. low plant and animal productivity) environments in northern Eurasia, only modern humans appear to have occupied the Arctic and Beringia. The critical variable was the capacity of modern humans for designing technology of unprecedented structural and functional complexity, including mechanical instruments and devices.

### (a) Neanderthals/Denisovans

The Neanderthals inhabited southwest Europe (including during glacial periods) where climates generally are mild and plant and animal productivity are relatively high, but their spatial/temporal distribution in eastern Europe and northern Asia, where climates are more continental and biological productivity lower, was limited. The Denisovans are found only in Asia. Both taxa were present in southwest Siberia (Altai Mountains) during the earlier Late Pleistocene. They appear to have been confined largely to places where natural shelters and wood were available [41,42].

Little is known about Denisovan anatomy or diet, but the Neanderthals are represented by a large sample of skeletal remains, which exhibit various anatomical adaptations to low temperature (e.g. thick chest, large head, shortened extremities) [41,43]. It may be assumed that their caloric demands were high, based on their body mass and climate setting. Stable isotope analyses and zooarchaeology indicate a high animal protein and fat diet (i.e. a northern diet) with an emphasis on large mammals [44]. Given their presence in southwest Europe during periods of extreme cold, low biological productivity may have been a more severe limiting factor than winter temperature. Despite their anatomical and dietary adaptations to northern environments, Neanderthal genetics reveal small populations with high inbreeding coefficients, subject to periodic local extinctions [41,45]. In the Altai region, a 2020 whole-genome analysis from *Chagyrskaya Cave* indicates an inbred population of less than 60 individuals [46].

### (b) Anatomically modern humans

New discoveries in southern France suggest an early presence of modern humans in southwest Europe during a warm interval before 50 ka [47], while evidence for their presence in the more continental regions of northern Eurasia post-dates cold HE 5 (less than 47 ka) [21,48]. A combination of dated human remains yielding ancient DNA (e.g. *Ust’-Ishim* in western Siberia) and dated artefact assemblages assigned to the Initial Upper Palaeolithic (IUP) industry (associated with modern human remains at *Bacho Kiro* in Bulgaria) suggests a rapid spread across central/eastern Europe and Siberia by 45 ka [21,48–50]. Modern humans were present, at least on a seasonal basis, in the European Arctic before 40 ka and probably on a year-round basis in arctic Siberia and Beringia by 33 ka [51,52]. The basal Eurasian lineages reflect significant genetic admixture from local Neanderthal/Denisovan populations [53,54].

Despite the genomic contribution from the local Neanderthal population, modern humans in northern Eurasia seem to have acquired few if any of the evolved anatomical adaptations to cold climate of the former. While only fragmentary skeletal remains have been recovered from time periods before 40 ka, complete skeletons from later periods before the LGM indicate retention of warm-climate anatomy (e.g. high brachial index) from lower latitudes, which would have rendered them susceptible to hypothermia and cold injury [55,56]. Stable isotope analyses and zooarchaeology reveal a northern diet comparable to that of the Neanderthals (i.e. significant niche overlap) but also a dietary breadth expanded to smaller vertebrates [57,58], while their genetics reflect adaptation to a diet high in polyunsaturated fatty acids (PUFAs) [59]. Palaeodemographic information for eastern Europe and arctic Siberia/Beringia suggest relatively large hunter–gatherer populations with little inbreeding [60,61].

Modern humans were an invasive species in northern Eurasia, hampered by an anatomical pattern better suited to the tropics. They nevertheless drove the local Neanderthal and Denisovan populations to extinction within a few thousand years, and occupied habitats and climate zones beyond the range of their predecessors. The explanation for this phenomenon lies in the fact that modern humans—including the basal Eurasian lineages—were equipped with most of the technologies found among recent hunter–gatherers in high latitudes [62].

Mechanical projectile weaponry is inferred from the morphometrics of Levallois points in the IUP and indicated by diagnostic impact fractures in southwest Europe approximately 45 ka [63,64]. Indirect evidence for snaring/trapping small mammals is reported from the East European Plain greater than 40 ka, while traces of mechanical rotary drills (which suggest fire-making technology) are found in both Eastern Europe (*Kostenki*) and southwest Siberia (*Denisova Cave*) in this time range. Evidence for sewn clothing (eyed needles) is dated to approximately 45 ka in the IUP level at Denisova Cave [62,65,66]. Reliable traces of artificial shelters date to 32 ka at the latest [67].

Equipped with these technologies, which appear to have been absent among Neanderthals and Denisovans (e.g. [68]), modern humans increased their foraging efficiency and success rate and harvested resources unavailable to the former in north Eurasian habitats where plant and animal productivity was low (especially during cold-climate periods). With tailored insulated clothing, artificial shelters, fire-making devices and alternative fuels (e.g. fresh bone), they not only survived extreme winter temperatures, but foraged effectively in cold weather and expanded their range into areas devoid of natural shelters and adequate wood fuel [48,62].

By 40 ka, palaeogenomics and archaeology indicate that regional populations had emerged or replaced the modern human meta-population in northern Eurasia. The East Asian lineage represented by the living Han population is identified at *Tianyuan Cave* in northern China at this time based on a whole-genome analysis of human remains [69]. The same lineage is present in the Amur River Basin at 33 ka and 19 ka (i.e. before and at the end of the LGM) [70]. It reflects early admixture with the Denisovans and is the primary ancestor (60–70%) of the Indigenous peoples of the Americas (arctic and non-arctic peoples) [61,71,72].

As described below, a suite of genetic adaptations to high latitudes (including low UV radiation) found today among both the arctic and non-arctic populations of the Western Hemisphere indicates that ancestral First Peoples inhabited the Arctic before the split between the two populations, which is estimated to have taken place during the LGM [73]. The ancestral group, which is designated *Ancient Palaeo-Siberians* (APS) in palaeogenomics, diverged from its East Asian parent lineage approximately 30 ka [95% CI 36.4–26.8 ka] [61,72]. APS is present in subarctic Siberia (59° North latitude) at *Khaiyrgas Cave* at 17 ka [74]. At this site, as well as at younger sites in Siberia and Beringia, APS is associated with a diagnostic set of stone artefacts comprising small wedge-shaped cores and microblades. These artefacts are found in older levels at Khaiyrgas Cave dating to the later LGM (approx. 24 ka) [74–76].

A second regional population with roots in western Eurasia (*Ancient North Eurasians* (ANE)) expanded into Northeast Asia before 30 ka and contributed to the East Asian genome and—both indirectly and directly—to the Native American genome. Genetic admixture with the west Eurasian lineage is evident at 34 ka in the woman from *Salkhit* in northern Mongolia [77]. The ANE population is present in southern Siberia during middle and late Upper Palaeolithic times at *Mal'ta* (24 ka) and *Afontova Gora* (17 ka), and their immediate ancestors (see below) are found in the Arctic and western Beringia as early as 33 ka [39,61,78].

## 5. The settlement of Beringia

### (a) Before the Last Glacial Maximum (35–26.5 ka)

Traces of a human presence in Beringia before 35 ka are limited and problematic, but given the evidence described above for occupation of the colder and drier parts of northern Eurasia 45–35 ka and the large mammal resources available on the exposed East Siberian Arctic Shelf during warm interstadials of this period, one or more episodes of modern human settlement in Beringia before 35 ka seems plausible, if not likely. The earliest well-dated archaeological sites and human remains are represented by a cluster of open-air sites near the mouth of the Yana River that have yielded thousands of stone and non-stone artefacts, and a large quantity of associated faunal remains, including steppe bison, reindeer, hare and mammoth. Calibration of a large sample of radiocarbon dates with the IntCal20 curve shows a concentration of ages around 34.5–31.5 ka, coinciding with two brief interstadials (*Greenland Interstadial 6* [GI 6] and GI 5) [21,39,52].

The Yana River sites are tied to exploitation of the GAP and probably represent a winter occupation on its southern upland margin. It is unclear whether the sites simply indicate a human presence in western arctic Beringia at this time or reflect a peak in human population numbers. Following the Mann *et al.* model [36], large mammal populations should have experienced one or more increases during the two brief interstadials. Whole-genome analyses of two human teeth recovered from the sites indicate a robust hunter–gatherer population with an estimated effective population size (*N*_e_) of 500 and low inbreeding coefficient (i.e. compare with Chagyrskaya Cave Neanderthal population described earlier) [61].

The ancient DNA analyses also reveal that the Yana River site occupants represent the west Eurasian lineage (designated *Ancient North Siberians* (ANS)) recently arrived in Northeast Asia. They carried a genetic adaptation to a northern diet (ancestral allele for expression of the *FADS1* gene, advantageous for a diet rich in PUFAs) which presumably reflects a shift from a low-latitude diet containing a relatively high proportion of plant foods [59]. They apparently did not develop any genetic adaptations to vitamin D deficiency related to low UV radiation during the winter months [70,79]. Conceivably, there were dietary sources of vitamin D in western arctic Beringia during the interstadials that preceded the LGM (e.g. mushrooms, eggs).

There is evidence for people in eastern Beringia in the form of biomarker data (faecal sterol molecular markers) from two lakes in arctic Alaska. Sediment cores from *Burial Lake* and *Lake E-5* in the Brooks Range yield recurring peaks of a high coprostanol : stigmastanol ratio (greater than 0.18) in the 34.5–31.5-ka time range and later. Although the faecal steroids are not species specific, the ratio values suggest a human presence relative to dominant background inputs of large herbivores. The cores also contain evidence for burning in the form of polycyclic aromatic hydrocarbon (PAH) fluxes and charcoal, another possible indicator of human activity in a region with limited lightning ignitions [80]. However, a 2021 field reconnaissance of Lake E-5 failed to turn up any archaeological remains (including evidence of Holocene occupation) [81].

Evidence for a human presence in mid-latitude North America (i.e. south of the Canadian ice sheets) and farther south before 15 ka is limited and controversial. The recently reported discovery of footprints in sediments dating to 23–21 ka on an ancient lakeshore in New Mexico provides an unusually strong case for people in the Western Hemisphere during the LGM [82], although the dating has been disputed (e.g. [83]). If people moved into mid-latitude North America before closure of the ice-free corridor, they conceivably represent either the west Eurasian lineage in Beringia before the LGM or the East Asian lineage in Beringia during the early LGM (see below). In either case, the size and long-term stability of any population in mid-latitude North America may have been severely constrained by the spatial–temporal dimensions of the ice-free corridor between 35 ka and its closure during the early LGM (25–23 ka or earlier).

Beringia itself may have been abandoned or have witnessed a severe decline in the human population after the GI 5 interstadial, which was followed by an episode of extreme cold climate (HE 3) at *ca* 30 ka [39]. The large-mammal biomass most likely decreased significantly at this time (and dietary sources of vitamin D may have disappeared altogether) due to increased aridity and reduced primary productivity.

### (b) During the Last Glacial Maximum (26.5–19 ka)

The LGM was a period of sustained cold and glacial re-advance in the Northern Hemisphere that reduced the land area of the Arctic to an arid zone between the North Eurasian and coalesced North American ice sheets (figure 3). An initial phase of extreme cold was interrupted by a brief interstadial (GI 2) at 24–23 ka, followed by another interval of low temperatures that were slightly milder than the initial phase [21]. Despite the extreme, sustained cryo-arid climate, which lowered plant and animal biomass, new research indicates that people were present in the colder and drier regions of northern Eurasia, including the central East European Plain and subarctic Siberia, although their numbers may have been significantly reduced [76,84].

A suite of high-latitude genetic adaptations (‘arctic signal’) found throughout the Indigenous peoples of the Western Hemisphere suggests that their immediate ancestors occupied the Arctic during the LGM [73,85]. The adaptations include the ancestral allele for *FADS1*, already common in the north Eurasian population, which became fixed not only in arctic and non-arctic First Peoples but also in related East Asian groups [59]. They also include a variant of *EDAR V370A*, which was under strong positive selection pressure during the LGM and appears to be an adaptation to vitamin D deficiency (due to low UV radiation) related to nursing infants [70,79]. In a recent paper, Niedbalski & Long attributed more than 20 000 high frequency alleles among living Native American populations to high latitude adaptation, including genes related to diet, cardiac processes and production of melanin in skin, hair and eyes [86]. The arctic signal also is evident in dental morphology: the distribution of three-rooted first molars in the Western Hemisphere suggests an arctic origin for the living First Peoples of mid-latitude North America and South America [87].

**Figure 4.**
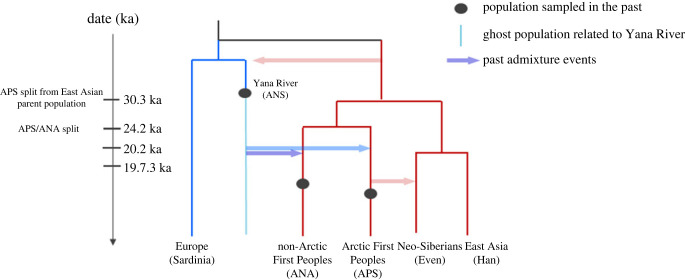
Palaeogenomic framework for the peopling of the Western Hemisphere, illustrating early split between west Eurasian lineage (left) and East Asian lineage (right) (adapted from Sikora *et al*. fig. 2b [61]).

As described earlier, the primary ancestor of living First Peoples (APS) diverged from its East Asian parent population approximately 30 ka [61,72]. The estimated time of divergence between APS and the First Peoples of mid-latitude North America and South America (designated *Ancient Native Americans* (ANA)) provides a minimum date for the presence of APS in the Arctic, based on the genetic ‘arctic signal’ in both populations. (In our view, the ANA designation is misleading because the APS population, which includes the living arctic peoples of North America, also contains Native American groups.) Sikora *et al.* concluded that the APS population split into two groups approximately 24 ka, one of which (designated *Ancient Beringians* (AB)) was the immediate source of the ANA population approximately 20–19 ka [61]. The AB are represented by aDNA from human remains in Alaska dating to 11.5 ka and 9 ka, respectively [72,88]. It now appears, however, that the ‘Ancient Beringians’ were a later offshoot of the North American ANA population [71]. Thus, the estimated time of APS/ANA divergence apparently lies somewhere between the GI 2 interstadial (approx. 24 ka) and the end of the LGM (19 ka) (figure 4).

The inferred presence of the APS population—or at least a portion of it—in the Arctic during the LGM has implications for the settlement history of Beringia. Most terrestrial habitat above the Arctic Circle was glaciated during 26.5–19 ka, excepting the region that lay between the north Eurasian and North American ice sheets. Most of this region was occupied by a dry plain that supported a relatively uniform steppe-tundra biome (i.e. the GAP) at least with respect to the fauna, as described previously [36,37,39]. We assume that any LGM human inhabitants of the GAP would have ranged widely across this environment, including areas that lie within conventional definitions of Beringia. We also assume, following the Mann *et al*. [36] model, that conditions for human occupation were most favourable during the brief GI 2 interstadial approximately 24 ka.

Evidence for a human presence in Beringia during the LGM is limited in comparison to the preceding interval [39,52]. The biomarker record from Burial Lake and Lake E-5 in eastern arctic Beringia suggests that people were present, at least periodically, during the LGM, however and the Burial Lake record exhibits a sustained peak for both the coprostanol : stigmastanol ratio and PAH fluxes at approximately 24–23 ka [80]. At *Bluefish Caves* (northern Yukon), humanly modified mammoth remains and radiocarbon-dated bones with tool cut marks suggest that humans occupied the site during the LGM [89], although their interpretation has been disputed [90].

### (c) After the Last Glacial Maximum (19–11 ka)

While climates warmed slightly after 19 ka, this interval was followed by another cold period (HE 1) during 15.7–14.5 ka [21]. Significant warming took place after HE 1, during the GI 1 (or *Bølling/Allerød*) interstadial 14.5–12.9 ka. Global sea levels began to rise gradually after approximately 16 ka although the BLB was not completely flooded until approximately 11 ka [91]. The dating of archaeological sites and human remains in mid-latitude North America shows that the ANA population dispersed rapidly in areas south of the Canadian ice sheets after 15 ka, reaching the southern tip of South America before 13 ka [2].

As described earlier, the relatively late opening of a traversable corridor between the retreating Cordilleran ice sheet and LIS (less than 14 ka) indicates that most, if not all, of the population that spread throughout the Western Hemisphere was derived from the NW Pacific coast. This conclusion helps explain the rapid growth and dispersal of the ANA population [14,92] because marine sediment cores from the North Pacific reveal a peak in marine productivity during the GI 1 interstadial [93]. Although the earliest traces of people on the NW Pacific coast currently date to *ca* 13 ka [94], they probably were present there before 15 ka.

The inferred presence of the ANA population on the NW Pacific coast before 15 ka has implications for the settlement history of Beringia. Before 15 ka, the NW Pacific was accessible to people in Beringia only from the southern BLB [24,25]. Because the narrow coastal zone restricted its human inhabitants to a diet based largely on marine resources, its earliest occupants probably arrived with a developed maritime economy (and some watercraft). Accordingly, we conclude that the ANA population probably adapted to coastal habitat on the southern BLB before 15 ka. We also conclude—based on the genetic ‘arctic signal’ found throughout arctic and non-arctic First Peoples—that the ANA population on the BLB was recently derived from the Arctic, most likely the GAP/arctic Beringia (not the NE Asian maritime zone) [73,86,95].

There is no supporting archaeological evidence for people on the southern BLB and we can only speculate on the process by which people from arctic Beringia developed a maritime economy on a subarctic coast (apparently abandoning the microblade technology of the APS along the way). The shift from terrestrial to coastal diet may have been driven by several factors. Population pressure on the inhabitants of the GAP (a consequence of elevated plant and animal productivity during the GI 2 interstadial approximately 24 ka?) conceivably promoted expansion into the subarctic zone, where—as discussed earlier—the large-mammal biomass and human carrying capacity of the terrestrial habitat must have been lower than that of the arctic plain (due to widespread tundra soil and peat formation). The marginal character of the terrestrial subarctic habitat would have encouraged greater dependance on marine resources along the southern coast (which began to retreat after 16 ka). Driftwood derived from a source (pine, alder) in the southern NW Pacific may have been available after 16 ka [96]. Perhaps cold HE 1, which began approximately 17 ka, was a catalyst for a shift to a marine diet at a time when terrestrial resources would have declined, while accelerated glacial run-off from eastern Beringia is thought to have fertilized waters on the southern coast [97].

A large and growing population on the NW Pacific coast during the GI 1 interstadial also has implications for the settlement history of Beringia. While most expansion of the ANA population apparently took place in mid-latitude North America and farther south, the geography of the narrow coastal zone probably promoted expansion northward (i.e. back into Beringia) as well. We suggest that most of the Beringian archaeological record for the GI 1 interstadial (14.5–12.9 ka), including the oldest occupations at *Ushki* (Kamchatka) and assemblages assigned to the *Nenana complex* in central Alaska [98], conceivably represent the northward expansion of the ANA population from the NW Pacific coast.

## 6. Conclusion and future research

We conclude that the distribution of North American ice sheets during and after the LGM was the critical variable in the peopling of the Western Hemisphere. Movement of both the APS and ANA populations (which diverged before 19 ka) from the interior of Beringia was blocked by the coalesced LIS and Cordilleran ice sheet until after 14 ka (by which time the ANA population already had dispersed widely in mid-latitude North America). Early deglaciation of the NW Pacific coast, which began approximately 18 ka, allowed the ANA population to occupy this region from the southern coast of the BLB before 15 ka and subsequently to disperse, from this region, throughout the unglaciated Western Hemisphere. An ice-free corridor between the two ice sheets was present before the LGM and may have allowed limited movement of people in the arctic or interior regions of Beringia to mid-latitude North America.

Our conclusion is bolstered by evidence from various spatial and temporal contexts in northern Eurasia that modern humans occupied most climate zones and habitats of the Northern Hemisphere after 45 ka, including the LGM during 26.5–19 ka [76,84]. Rather than presenting an *ecological barrier*, the large mammal populations of the GAP—much of which lies within arctic Beringia—probably attracted modern humans to high latitudes, although genetic adaptation to low UV radiation, especially in the absence of dietary sources of vitamin D, may have been an important factor in the settlement history of the GAP [79]. Marine resources along the southern coast of Beringia and adjoining areas of the NW Pacific, which became especially rich after 14.5 ka, also must have attracted human settlement.

We conclude that the variation among living First Peoples is more likely to be a product of regional differentiation of populations after their divergence from their source population in Northeast Asia, and not the result of multiple migrations to the Western Hemisphere [10,11]. This conclusion is based on a synthesis of recent research in geology and genetics suggesting that: (a) the ANA population dispersed from the NW Pacific coast, which must have been accessed from the southern BLB; and (b) the immediate ancestors of the ANA population (i.e. APS) occupied the Arctic, including arctic Beringia, before the APS/ANA split. We conclude that the primary division among living First Peoples (i.e. arctic versus non-arctic) is the result of an event that took place in Beringia before 15 ka, i.e. divergence of arctic interior (GAP) and subarctic coastal (southern BLB) adaptations and peoples.

Although these conclusions are broadly consistent with the 2007 ‘Beringian standstill’ model, which postulated an extended period of isolation for the Native American founder population in Beringia [13–15], we now view the term ‘standstill’ as a mischaracterization of what probably took place during and after the LGM. The founder population did not retreat into a refugium during the LGM but expanded into an immense arctic plain where extreme climate and lowered sea level promoted steppe-tundra flora and fauna. During or after the LGM, the population spread into a less productive subarctic environment and eventually developed a northern coastal economy, which facilitated further expansion into the NW Pacific and massive population growth and dispersal during the final interstadial of the Pleistocene.

By linking the divergence of arctic and non-arctic First Peoples to development of a coastal adaptation in southern Beringia that subsequently gave rise to the large NW Pacific coast population, our conclusions may be reconciled with the multiple migration model, at least in part. The early NW Pacific coast peoples conceivably represent the ancestral Dené [99], who constitute a major linguistic and biological group within the Indigenous peoples of the Western Hemisphere [8–11,87]. The multiple migrations of the 1986 ‘three-wave model’ (see introduction) may be equated with the three steps of geographical expansion postulated here (i.e. Beringia, NW Pacific coast and mid-latitude North America and South America).

In our view, the most promising avenue of future research on the questions addressed in this review lies in those areas of northeastern Siberia and northern Alaska/Yukon that represent the former southern and eastern upland margins of the GAP, including islands in the East Siberian Sea. Ongoing efforts to reconstruct fluctuations in large herbivore populations will shed light on the resources available to people on the GAP before, during, and after the LGM [36,40].

We are pursuing field research on both sides of the Bering Strait, including western arctic Beringia, which yields evidence for the presence of the west Eurasian lineage before the LGM, but has yet to produce definitive evidence for the East Asian lineage greater than 10 ka, although a credible archaeological proxy for APS (microblades) dates to approximately 15 ka [39,52]). New research at Bluefish Caves (northern Yukon) should resolve uncertainties regarding the stratigraphic context and dating of the stone artefacts and modified bones [89]. Another focus of research in eastern Beringia is the small number of lakes (e.g. Burial Lake) that did not evaporate during the LGM, several of which have yielded biomarker evidence for people before and during the LGM [80,81].

## Data Availability

This article has no additional data.
